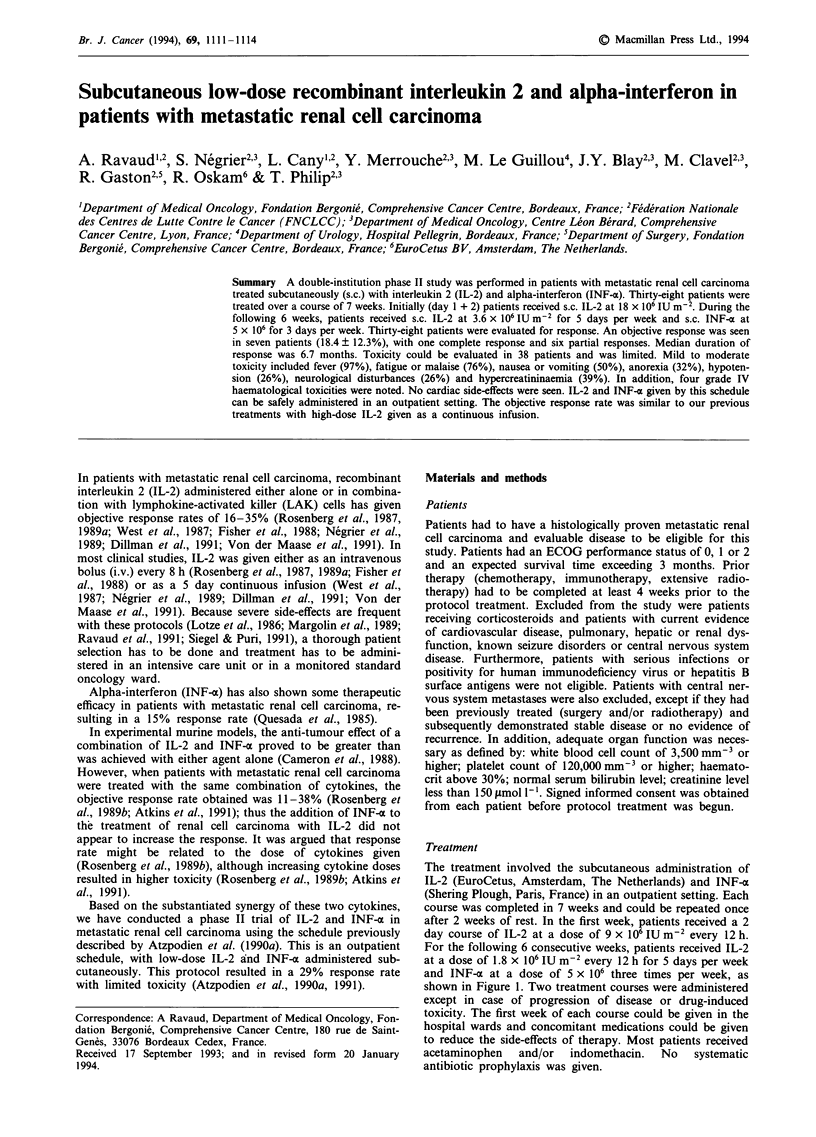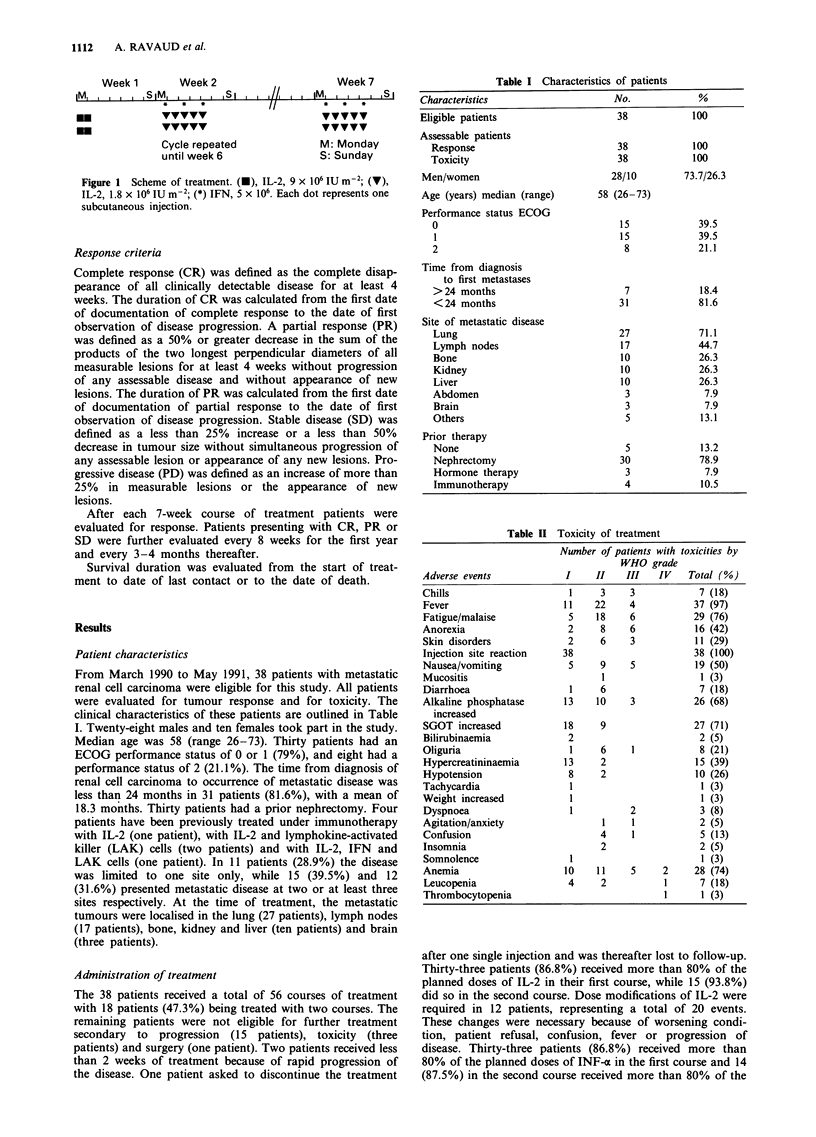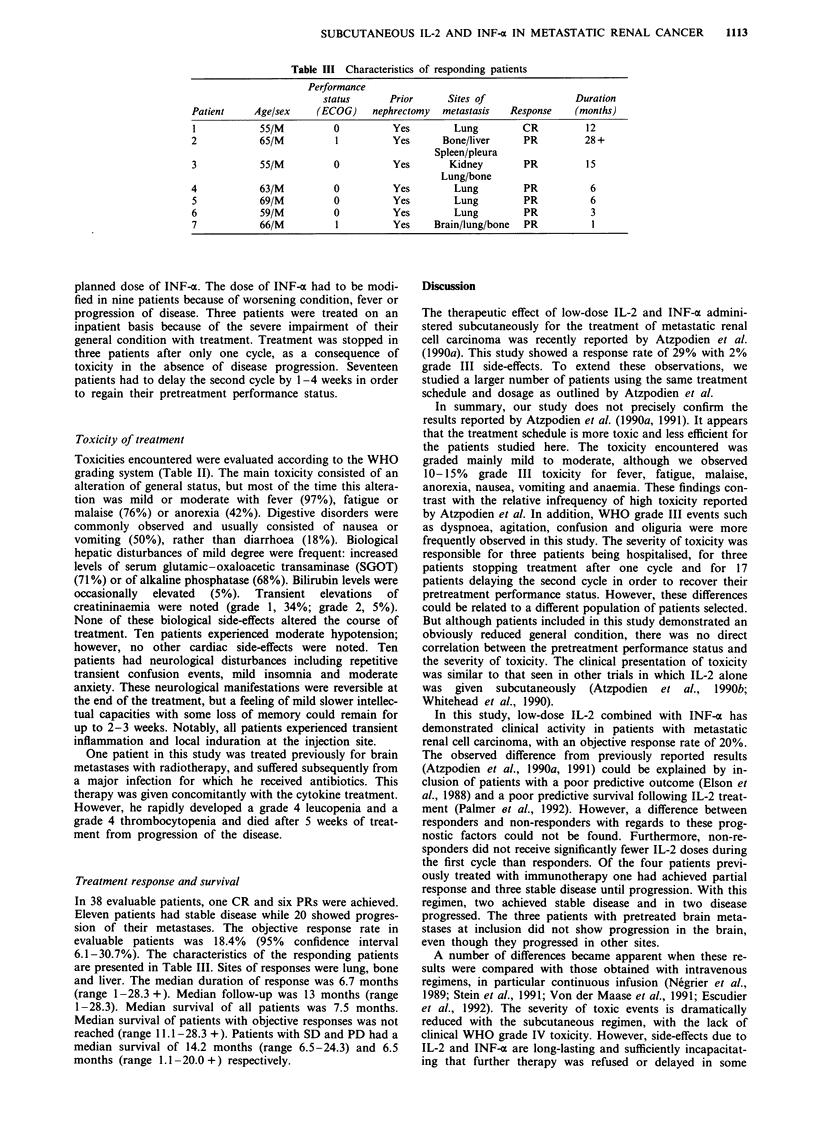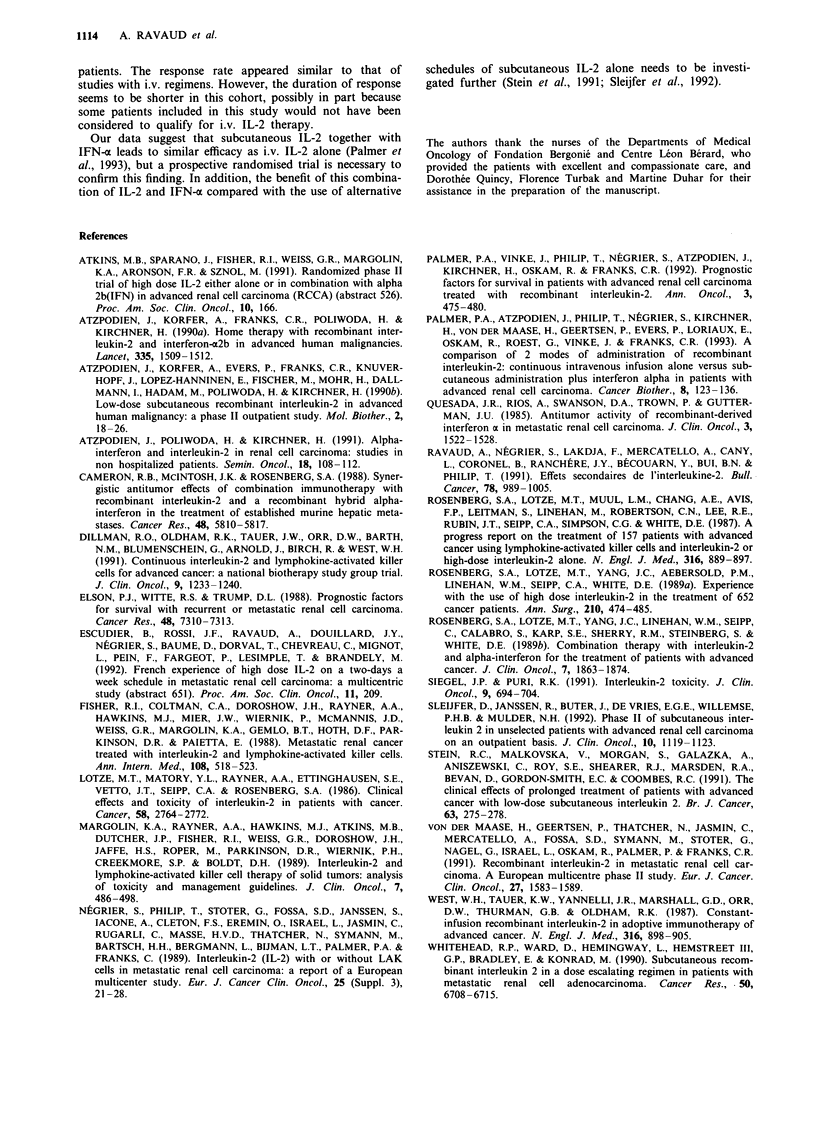# Subcutaneous low-dose recombinant interleukin 2 and alpha-interferon in patients with metastatic renal cell carcinoma.

**DOI:** 10.1038/bjc.1994.218

**Published:** 1994-06

**Authors:** A. Ravaud, S. Négrier, L. Cany, Y. Merrouche, M. Le Guillou, J. Y. Blay, M. Clavel, R. Gaston, R. Oskam, T. Philip

**Affiliations:** Department of Medical Oncology, Foundation Bergonié, Comprehensive Cancer Centre, Bordeaux, France.

## Abstract

A double-institution phase II study was performed in patients with metastatic renal cell carcinoma treated subcutaneously (s.c.) with interleukin 2 (IL-2) and alpha-interferon (INF-alpha). Thirty-eight patients were treated over a course of 7 weeks. Initially (day 1 + 2) patients received s.c. IL-2 at 18 x 10(6) IU m-2. During the following 6 weeks, patients received s.c. IL-2 at 3.6 x 10(6) IU m-2 for 5 days per week and s.c. INF-alpha at 5 x 10(6) for 3 days per week. Thirty-eight patients were evaluated for response. An objective response was seen in seven patients (18.4 +/- 12.3%), with one complete response and six partial responses. Median duration of response was 6.7 months. Toxicity could be evaluated in 38 patients and was limited. Mild to moderate toxicity included fever (97%), fatigue or malaise (76%), nausea or vomiting (50%), anorexia (32%), hypotension (26%), neurological disturbances (26%) and hypercreatininaemia (39%). In addition, four grade IV haematological toxicities were noted. No cardiac side-effects were seen. IL-2 and INF-alpha given by this schedule can be safely administered in an outpatient setting. The objective response rate was similar to our previous treatments with high-dose IL-2 given as a continuous infusion.


*Br. J. Cancer* (1994), **69**, 1111–1114 © Macmillan Press Ltd., 1994

### Subcutaneous low-dose recombinant interleukin 2 and alpha-interferon in patients with metastatic renal cell carcinoma

A. Ravaud[1,2], S. Négrier[2,3], L. Cany[1,2], Y. Merrouche[2,3], M. Le Guillou[4], J.Y. Blay[2,3], M. Clavel[2,3], R. Gaston[2,5], R. Oskam[6] & T. Philip[2,3]

[1]*Department of Medical Oncology, Fondation Bergonié, Comprehensive Cancer Centre, Bordeaux, France;* [2]*Fédération Nationale des Centres de Lutte Contre le Cancer (FNCLCC);* [3]*Department of Medical Oncology, Centre Léon Bérard, Comprehensive Cancer Centre, Lyon, France;* [4]*Department of Urology, Hospital Pellegrin, Bordeaux, France;* [5]*Department of Surgery, Fondation Bergonié, Comprehensive Cancer Centre, Bordeaux, France;* [6]*EuroCetus BV, Amsterdam, The Netherlands.*

**Summary** A double-institution phase II study was performed in patients with metastatic renal cell carcinoma treated subcutaneously (s.c.) with interleukin 2 (IL-2) and alpha-interferon (INF-α). Thirty-eight patients were treated over a course of 7 weeks. Initially (day 1 + 2) patients received s.c. IL-2 at $18 \times 10^6$ IU $m^{-2}$. During the following 6 weeks, patients received s.c. IL-2 at $3.6 \times 10^6$ IU $m^{-2}$ for 5 days per week and s.c. INF-α at $5 \times 10^6$ for 3 days per week. Thirty-eight patients were evaluated for response. An objective response was seen in seven patients (18.4 ± 12.3%), with one complete response and six partial responses. Median duration of response was 6.7 months. Toxicity could be evaluated in 38 patients and was limited. Mild to moderate toxicity included fever (97%), fatigue or malaise (76%), nausea or vomiting (50%), anorexia (32%), hypotension (26%), neurological disturbances (26%) and hypercreatininaemia (39%). In addition, four grade IV haematological toxicities were noted. No cardiac side-effects were seen. IL-2 and INF-α given by this schedule can be safely administered in an outpatient setting. The objective response rate was similar to our previous treatments with high-dose IL-2 given as a continuous infusion.

In patients with metastatic renal cell carcinoma, recombinant interleukin 2 (IL-2) administered either alone or in combination with lymphokine-activated killer (LAK) cells has given objective response rates of 16–35% (Rosenberg *et al.*, 1987, 1989*a*; West *et al.*, 1987; Fisher *et al.*, 1988; Négrier *et al.*, 1989; Dillman *et al.*, 1991; Von der Maase *et al.*, 1991). In most clinical studies, IL-2 was given either as an intravenous bolus (i.v.) every 8 h (Rosenberg *et al.*, 1987, 1989*a*; Fisher *et al.*, 1988) or as a 5 day continuous infusion (West *et al.*, 1987; Négrier *et al.*, 1989; Dillman *et al.*, 1991; Von der Maase *et al.*, 1991). Because severe side-effects are frequent with these protocols (Lotze *et al.*, 1986; Margolin *et al.*, 1989; Ravaud *et al.*, 1991; Siegel & Puri, 1991), a thorough patient selection has to be done and treatment has to be administered in an intensive care unit or in a monitored standard oncology ward.

Alpha-interferon (INF-α) has also shown some therapeutic efficacy in patients with metastatic renal cell carcinoma, resulting in a 15% response rate (Quesada *et al.*, 1985).

In experimental murine models, the anti-tumour effect of a combination of IL-2 and INF-α proved to be greater than was achieved with either agent alone (Cameron *et al.*, 1988). However, when patients with metastatic renal cell carcinoma were treated with the same combination of cytokines, the objective response rate obtained was 11–38% (Rosenberg *et al.*, 1989*b*; Atkins *et al.*, 1991); thus the addition of INF-α to the treatment of renal cell carcinoma with IL-2 did not appear to increase the response. It was argued that response rate might be related to the dose of cytokines given (Rosenberg *et al.*, 1989*b*), although increasing cytokine doses resulted in higher toxicity (Rosenberg *et al.*, 1989*b*; Atkins *et al.*, 1991).

Based on the substantiated synergy of these two cytokines, we have conducted a phase II trial of IL-2 and INF-α in metastatic renal cell carcinoma using the schedule previously described by Atzpodien *et al.* (1990*a*). This is an outpatient schedule, with low-dose IL-2 and INF-α administered subcutaneously. This protocol resulted in a 29% response rate with limited toxicity (Atzpodien *et al.*, 1990*a*, 1991).

Correspondence: A Ravaud, Department of Medical Oncology, Fondation Bergonié, Comprehensive Cancer Centre, 180 rue de Saint-Genès, 33076 Bordeaux Cedex, France.
Received 17 September 1993; and in revised form 20 January 1994.

### Materials and methods

### *Patients*

Patients had to have a histologically proven metastatic renal cell carcinoma and evaluable disease to be eligible for this study. Patients had an ECOG performance status of 0, 1 or 2 and an expected survival time exceeding 3 months. Prior therapy (chemotherapy, immunotherapy, extensive radiotherapy) had to be completed at least 4 weeks prior to the protocol treatment. Excluded from the study were patients receiving corticosteroids and patients with current evidence of cardiovascular disease, pulmonary, hepatic or renal dysfunction, known seizure disorders or central nervous system disease. Furthermore, patients with serious infections or positivity for human immunodeficiency virus or hepatitis B surface antigens were not eligible. Patients with central nervous system metastases were also excluded, except if they had been previously treated (surgery and/or radiotherapy) and subsequently demonstrated stable disease or no evidence of recurrence. In addition, adequate organ function was necessary as defined by: white blood cell count of 3,500 $mm^{-3}$ or higher; platelet count of 120,000 $mm^{-3}$ or higher; haematocrit above 30%; normal serum bilirubin level; creatinine level less than 150 $\mu mol\ l^{-1}$. Signed informed consent was obtained from each patient before protocol treatment was begun.

### *Treatment*

The treatment involved the subcutaneous administration of IL-2 (EuroCetus, Amsterdam, The Netherlands) and INF-α (Shering Plough, Paris, France) in an outpatient setting. Each course was completed in 7 weeks and could be repeated once after 2 weeks of rest. In the first week, patients received a 2 day course of IL-2 at a dose of $9 \times 10^6$ IU $m^{-2}$ every 12 h. For the following 6 consecutive weeks, patients received IL-2 at a dose of $1.8 \times 10^6$ IU $m^{-2}$ every 12 h for 5 days per week and INF-α at a dose of $5 \times 10^6$ three times per week, as shown in Figure 1. Two treatment courses were administered except in case of progression of disease or drug-induced toxicity. The first week of each course could be given in the hospital wards and concomitant medications could be given to reduce the side-effects of therapy. Most patients received acetaminophen and/or indomethacin. No systematic antibiotic prophylaxis was given.

**Figure 1** Scheme of treatment. (■), IL-2, $9 \times 10^6$ IU $m^{-2}$; (▼), IL-2, $1.8 \times 10^6$ IU $m^{-2}$; (*) IFN, $5 \times 10^6$. Each dot represents one subcutaneous injection.

*Response criteria*

Complete response (CR) was defined as the complete disappearance of all clinically detectable disease for at least 4 weeks. The duration of CR was calculated from the first date of documentation of complete response to the date of first observation of disease progression. A partial response (PR) was defined as a 50% or greater decrease in the sum of the products of the two longest perpendicular diameters of all measurable lesions for at least 4 weeks without progression of any assessable disease and without appearance of new lesions. The duration of PR was calculated from the first date of documentation of partial response to the date of first observation of disease progression. Stable disease (SD) was defined as a less than 25% increase or a less than 50% decrease in tumour size without simultaneous progression of any assessable lesion or appearance of any new lesions. Progressive disease (PD) was defined as an increase of more than 25% in measurable lesions or the appearance of new lesions.

After each 7-week course of treatment patients were evaluated for response. Patients presenting with CR, PR or SD were further evaluated every 8 weeks for the first year and every 3–4 months thereafter.

Survival duration was evaluated from the start of treatment to date of last contact or to the date of death.

### Results

*Patient characteristics*

From March 1990 to May 1991, 38 patients with metastatic renal cell carcinoma were eligible for this study. All patients were evaluated for tumour response and for toxicity. The clinical characteristics of these patients are outlined in Table I. Twenty-eight males and ten females took part in the study. Median age was 58 (range 26–73). Thirty patients had an ECOG performance status of 0 or 1 (79%), and eight had a performance status of 2 (21.1%). The time from diagnosis of renal cell carcinoma to occurrence of metastatic disease was less than 24 months in 31 patients (81.6%), with a mean of 18.3 months. Thirty patients had a prior nephrectomy. Four patients have been previously treated under immunotherapy with IL-2 (one patient), with IL-2 and lymphokine-activated killer (LAK) cells (two patients) and with IL-2, IFN and LAK cells (one patient). In 11 patients (28.9%) the disease was limited to one site only, while 15 (39.5%) and 12 (31.6%) presented metastatic disease at two or at least three sites respectively. At the time of treatment, the metastatic tumours were localised in the lung (27 patients), lymph nodes (17 patients), bone, kidney and liver (ten patients) and brain (three patients).

*Administration of treatment*

The 38 patients received a total of 56 courses of treatment with 18 patients (47.3%) being treated with two courses. The remaining patients were not eligible for further treatment secondary to progression (15 patients), toxicity (three patients) and surgery (one patient). Two patients received less than 2 weeks of treatment because of rapid progression of the disease. One patient asked to discontinue the treatment after one single injection and was thereafter lost to follow-up. Thirty-three patients (86.8%) received more than 80% of the planned doses of IL-2 in their first course, while 15 (93.8%) did so in the second course. Dose modifications of IL-2 were required in 12 patients, representing a total of 20 events. These changes were necessary because of worsening condition, patient refusal, confusion, fever or progression of disease. Thirty-three patients (86.8%) received more than 80% of the planned doses of INF-α in the first course and 14 (87.5%) in the second course received more than 80% of the

**Table I** Characteristics of patients

| *Characteristics* | *No.* | *%* |
|---|---|---|
| Eligible patients | 38 | 100 |
| Assessable patients | | |
| Response | 38 | 100 |
| Toxicity | 38 | 100 |
| Men/women | 28/10 | 73.7/26.3 |
| Age (years) median (range) | 58 (26–73) | |
| Performance status ECOG | | |
| 0 | 15 | 39.5 |
| 1 | 15 | 39.5 |
| 2 | 8 | 21.1 |
| Time from diagnosis to first metastases | | |
| >24 months | 7 | 18.4 |
| <24 months | 31 | 81.6 |
| Site of metastatic disease | | |
| Lung | 27 | 71.1 |
| Lymph nodes | 17 | 44.7 |
| Bone | 10 | 26.3 |
| Kidney | 10 | 26.3 |
| Liver | 10 | 26.3 |
| Abdomen | 3 | 7.9 |
| Brain | 3 | 7.9 |
| Others | 5 | 13.1 |
| Prior therapy | | |
| None | 5 | 13.2 |
| Nephrectomy | 30 | 78.9 |
| Hormone therapy | 3 | 7.9 |
| Immunotherapy | 4 | 10.5 |

**Table II** Toxicity of treatment

| | *Number of patients with toxicities by WHO grade* | | | | |
|---|---|---|---|---|---|
| *Adverse events* | *I* | *II* | *III* | *IV* | *Total (%)* |
| Chills | 1 | 3 | 3 | | 7 (18) |
| Fever | 11 | 22 | 4 | | 37 (97) |
| Fatigue/malaise | 5 | 18 | 6 | | 29 (76) |
| Anorexia | 2 | 8 | 6 | | 16 (42) |
| Skin disorders | 2 | 6 | 3 | | 11 (29) |
| Injection site reaction | 38 | | | | 38 (100) |
| Nausea/vomiting | 5 | 9 | 5 | | 19 (50) |
| Mucositis | | 1 | | | 1 (3) |
| Diarrhoea | 1 | 6 | | | 7 (18) |
| Alkaline phosphatase increased | 13 | 10 | 3 | | 26 (68) |
| SGOT increased | 18 | 9 | | | 27 (71) |
| Bilirubinaemia | 2 | | | | 2 (5) |
| Oliguria | 1 | 6 | 1 | | 8 (21) |
| Hypercreatininaemia | 13 | 2 | | | 15 (39) |
| Hypotension | 8 | 2 | | | 10 (26) |
| Tachycardia | 1 | | | | 1 (3) |
| Weight increased | 1 | | | | 1 (3) |
| Dyspnoea | 1 | | 2 | | 3 (8) |
| Agitation/anxiety | | 1 | 1 | | 2 (5) |
| Confusion | | 4 | 1 | | 5 (13) |
| Insomnia | | 2 | | | 2 (5) |
| Somnolence | 1 | | | | 1 (3) |
| Anemia | 10 | 11 | 5 | 2 | 28 (74) |
| Leucopenia | 4 | 2 | | 1 | 7 (18) |
| Thrombocytopenia | | | | 1 | 1 (3) |

**Table III** Characteristics of responding patients

| Patient | Age/sex | Performance status (ECOG) | Prior nephrectomy | Sites of metastasis | Response | Duration (months) |
|---|---|---|---|---|---|---|
| 1 | 55/M | 0 | Yes | Lung | CR | 12 |
| 2 | 65/M | 1 | Yes | Bone/liver Spleen/pleura | PR | 28+ |
| 3 | 55/M | 0 | Yes | Kidney Lung/bone | PR | 15 |
| 4 | 63/M | 0 | Yes | Lung | PR | 6 |
| 5 | 69/M | 0 | Yes | Lung | PR | 6 |
| 6 | 59/M | 0 | Yes | Lung | PR | 3 |
| 7 | 66/M | 1 | Yes | Brain/lung/bone | PR | 1 |

planned dose of INF-α. The dose of INF-α had to be modified in nine patients because of worsening condition, fever or progression of disease. Three patients were treated on an inpatient basis because of the severe impairment of their general condition with treatment. Treatment was stopped in three patients after only one cycle, as a consequence of toxicity in the absence of disease progression. Seventeen patients had to delay the second cycle by 1–4 weeks in order to regain their pretreatment performance status.

### *Toxicity of treatment*

Toxicities encountered were evaluated according to the WHO grading system (Table II). The main toxicity consisted of an alteration of general status, but most of the time this alteration was mild or moderate with fever (97%), fatigue or malaise (76%) or anorexia (42%). Digestive disorders were commonly observed and usually consisted of nausea or vomiting (50%), rather than diarrhoea (18%). Biological hepatic disturbances of mild degree were frequent: increased levels of serum glutamic–oxaloacetic transaminase (SGOT) (71%) or of alkaline phosphatase (68%). Bilirubin levels were occasionally elevated (5%). Transient elevations of creatininaemia were noted (grade 1, 34%; grade 2, 5%). None of these biological side-effects altered the course of treatment. Ten patients experienced moderate hypotension; however, no other cardiac side-effects were noted. Ten patients had neurological disturbances including repetitive transient confusion events, mild insomnia and moderate anxiety. These neurological manifestations were reversible at the end of the treatment, but a feeling of mild slower intellectual capacities with some loss of memory could remain for up to 2–3 weeks. Notably, all patients experienced transient inflammation and local induration at the injection site.

One patient in this study was treated previously for brain metastases with radiotherapy, and suffered subsequently from a major infection for which he received antibiotics. This therapy was given concomitantly with the cytokine treatment. However, he rapidly developed a grade 4 leucopenia and a grade 4 thrombocytopenia and died after 5 weeks of treatment from progression of the disease.

### *Treatment response and survival*

In 38 evaluable patients, one CR and six PRs were achieved. Eleven patients had stable disease while 20 showed progression of their metastases. The objective response rate in evaluable patients was 18.4% (95% confidence interval 6.1–30.7%). The characteristics of the responding patients are presented in Table III. Sites of responses were lung, bone and liver. The median duration of response was 6.7 months (range 1–28.3 +). Median follow-up was 13 months (range 1–28.3). Median survival of all patients was 7.5 months. Median survival of patients with objective responses was not reached (range 11.1–28.3 +). Patients with SD and PD had a median survival of 14.2 months (range 6.5–24.3) and 6.5 months (range 1.1–20.0 +) respectively.

### Discussion

The therapeutic effect of low-dose IL-2 and INF-α administered subcutaneously for the treatment of metastatic renal cell carcinoma was recently reported by Atzpodien *et al.* (1990*a*). This study showed a response rate of 29% with 2% grade III side-effects. To extend these observations, we studied a larger number of patients using the same treatment schedule and dosage as outlined by Atzpodien *et al.*

In summary, our study does not precisely confirm the results reported by Atzpodien *et al.* (1990*a*, 1991). It appears that the treatment schedule is more toxic and less efficient for the patients studied here. The toxicity encountered was graded mainly mild to moderate, although we observed 10–15% grade III toxicity for fever, fatigue, malaise, anorexia, nausea, vomiting and anaemia. These findings contrast with the relative infrequency of high toxicity reported by Atzpodien *et al.* In addition, WHO grade III events such as dyspnoea, agitation, confusion and oliguria were more frequently observed in this study. The severity of toxicity was responsible for three patients being hospitalised, for three patients stopping treatment after one cycle and for 17 patients delaying the second cycle in order to recover their pretreatment performance status. However, these differences could be related to a different population of patients selected. But although patients included in this study demonstrated an obviously reduced general condition, there was no direct correlation between the pretreatment performance status and the severity of toxicity. The clinical presentation of toxicity was similar to that seen in other trials in which IL-2 alone was given subcutaneously (Atzpodien *et al.*, 1990*b*; Whitehead *et al.*, 1990).

In this study, low-dose IL-2 combined with INF-α has demonstrated clinical activity in patients with metastatic renal cell carcinoma, with an objective response rate of 20%. The observed difference from previously reported results (Atzpodien *et al.*, 1990*a*, 1991) could be explained by inclusion of patients with a poor predictive outcome (Elson *et al.*, 1988) and a poor predictive survival following IL-2 treatment (Palmer *et al.*, 1992). However, a difference between responders and non-responders with regards to these prognostic factors could not be found. Furthermore, non-responders did not receive significantly fewer IL-2 doses during the first cycle than responders. Of the four patients previously treated with immunotherapy one had achieved partial response and three stable disease until progression. With this regimen, two achieved stable disease and in two disease progressed. The three patients with pretreated brain metastases at inclusion did not show progression in the brain, even though they progressed in other sites.

A number of differences became apparent when these results were compared with those obtained with intravenous regimens, in particular continuous infusion (Négrier *et al.*, 1989; Stein *et al.*, 1991; Von der Maase *et al.*, 1991; Escudier *et al.*, 1992). The severity of toxic events is dramatically reduced with the subcutaneous regimen, with the lack of clinical WHO grade IV toxicity. However, side-effects due to IL-2 and INF-α are long-lasting and sufficiently incapacitating that further therapy was refused or delayed in some patients. The response rate appeared similar to that of studies with i.v. regimens. However, the duration of response seems to be shorter in this cohort, possibly in part because some patients included in this study would not have been considered to qualify for i.v. IL-2 therapy.

Our data suggest that subcutaneous IL-2 together with IFN-α leads to similar efficacy as i.v. IL-2 alone (Palmer *et al.*, 1993), but a prospective randomised trial is necessary to confirm this finding. In addition, the benefit of this combination of IL-2 and IFN-α compared with the use of alternative schedules of subcutaneous IL-2 alone needs to be investigated further (Stein *et al.*, 1991; Sleijfer *et al.*, 1992).

The authors thank the nurses of the Departments of Medical Oncology of Fondation Bergonié and Centre Léon Bérard, who provided the patients with excellent and compassionate care, and Dorothée Quincy, Florence Turbak and Martine Duhar for their assistance in the preparation of the manuscript.

### References

ATKINS, M.B., SPARANO, J., FISHER, R.I., WEISS, G.R., MARGOLIN, K.A., ARONSON, F.R. & SZNOL, M. (1991). Randomized phase II trial of high dose IL-2 either alone or in combination with alpha 2b(IFN) in advanced renal cell carcinoma (RCCA) (abstract 526). *Proc. Am. Soc. Clin. Oncol.*, **10**, 166.

ATZPODIEN, J., KORFER, A., FRANKS, C.R., POLIWODA, H. & KIRCHNER, H. (1990*a*). Home therapy with recombinant interleukin-2 and interferon-α2b in advanced human malignancies. *Lancet*, **335**, 1509–1512.

ATZPODIEN, J., KORFER, A., EVERS, P., FRANKS, C.R., KNUVERHOPF, J., LOPEZ-HANNINEN, E., FISCHER, M., MOHR, H., DALLMANN, I., HADAM, M., POLIWODA, H. & KIRCHNER, H. (1990*b*). Low-dose subcutaneous recombinant interleukin-2 in advanced human malignancy: a phase II outpatient study. *Mol. Biother.*, **2**, 18–26.

ATZPODIEN, J., POLIWODA, H. & KIRCHNER, H. (1991). Alpha-interferon and interleukin-2 in renal cell carcinoma: studies in non hospitalized patients. *Semin. Oncol.*, **18**, 108–112.

CAMERON, R.B., MCINTOSH, J.K. & ROSENBERG, S.A. (1988). Synergistic antitumor effects of combination immunotherapy with recombinant interleukin-2 and a recombinant hybrid alpha-interferon in the treatment of established murine hepatic metastases. *Cancer Res.*, **48**, 5810–5817.

DILLMAN, R.O., OLDHAM, R.K., TAUER, J.W., ORR, D.W., BARTH, N.M., BLUMENSCHEIN, G., ARNOLD, J., BIRCH, R. & WEST, W.H. (1991). Continuous interleukin-2 and lymphokine-activated killer cells for advanced cancer: a national biotherapy study group trial. *J. Clin. Oncol.*, **9**, 1233–1240.

ELSON, P.J., WITTE, R.S. & TRUMP, D.L. (1988). Prognostic factors for survival with recurrent or metastatic renal cell carcinoma. *Cancer Res.*, **48**, 7310–7313.

ESCUDIER, B., ROSSI, J.F., RAVAUD, A., DOUILLARD, J.Y., NÉGRIER, S., BAUME, D., DORVAL, T., CHEVREAU, C., MIGNOT, L., PEIN, F., FARGEOT, P., LESIMPLE, T. & BRANDELY, M. (1992). French experience of high dose IL-2 on a two-days a week schedule in metastatic renal cell carcinoma: a multicentric study (abstract 651). *Proc. Am. Soc. Clin. Oncol.*, **11**, 209.

FISHER, R.I., COLTMAN, C.A., DOROSHOW, J.H., RAYNER, A.A., HAWKINS, M.J., MIER, J.W., WIERNIK, P., MCMANNIS, J.D., WEISS, G.R., MARGOLIN, K.A., GEMLO, B.T., HOTH, D.F., PARKINSON, D.R. & PAIETTA, E. (1988). Metastatic renal cancer treated with interleukin-2 and lymphokine-activated killer cells. *Ann. Intern. Med.*, **108**, 518–523.

LOTZE, M.T., MATORY, Y.L., RAYNER, A.A., ETTINGHAUSEN, S.E., VETTO, J.T., SEIPP, C.A. & ROSENBERG, S.A. (1986). Clinical effects and toxicity of interleukin-2 in patients with cancer. *Cancer*, **58**, 2764–2772.

MARGOLIN, K.A., RAYNER, A.A., HAWKINS, M.J., ATKINS, M.B., DUTCHER, J.P., FISHER, R.I., WEISS, G.R., DOROSHOW, J.H., JAFFE, H.S., ROPER, M., PARKINSON, D.R., WIERNIK, P.H., CREEKMORE, S.P. & BOLDT, D.H. (1989). Interleukin-2 and lymphokine-activated killer cell therapy of solid tumors: analysis of toxicity and management guidelines. *J. Clin. Oncol.*, **7**, 486–498.

NÉGRIER, S., PHILIP, T., STOTER, G., FOSSA, S.D., JANSSEN, S., IACONE, A., CLETON, F.S., EREMIN, O., ISRAEL, L., JASMIN, C., RUGARLI, C., MASSE, H.V.D., THATCHER, N., SYMANN, M., BARTSCH, H.H., BERGMANN, L., BIJMAN, L.T., PALMER, P.A. & FRANKS, C. (1989). Interleukin-2 (IL-2) with or without LAK cells in metastatic renal cell carcinoma: a report of a European multicenter study. *Eur. J. Cancer Clin. Oncol.*, **25** (Suppl. 3), 21–28.

PALMER, P.A., VINKE, J., PHILIP, T., NÉGRIER, S., ATZPODIEN, J., KIRCHNER, H., OSKAM, R. & FRANKS, C.R. (1992). Prognostic factors for survival in patients with advanced renal cell carcinoma treated with recombinant interleukin-2. *Ann. Oncol.*, **3**, 475–480.

PALMER, P.A., ATZPODIEN, J., PHILIP, T., NÉGRIER, S., KIRCHNER, H., VON DER MAASE, H., GEERTSEN, P., EVERS, P., LORIAUX, E., OSKAM, R., ROEST, G., VINKE, J. & FRANKS, C.R. (1993). A comparison of 2 modes of administration of recombinant interleukin-2: continuous intravenous infusion alone versus subcutaneous administration plus interferon alpha in patients with advanced renal cell carcinoma. *Cancer Biother.*, **8**, 123–136.

QUESADA, J.R., RIOS, A., SWANSON, D.A., TROWN, P. & GUTTERMAN, J.U. (1985). Antitumor activity of recombinant-derived interferon α in metastatic renal cell carcinoma. *J. Clin. Oncol.*, **3**, 1522–1528.

RAVAUD, A., NÉGRIER, S., LAKDJA, F., MERCATELLO, A., CANY, L., CORONEL, B., RANCHÉRE, J.Y., BÉCOUARN, Y., BUI, B.N. & PHILIP, T. (1991). Effets secondaires de l'interleukine-2. *Bull. Cancer*, **78**, 989–1005.

ROSENBERG, S.A., LOTZE, M.T., MUUL, L.M., CHANG, A.E., AVIS, F.P., LEITMAN, S., LINEHAN, M., ROBERTSON, C.N., LEE, R.E., RUBIN, J.T., SEIPP, C.A., SIMPSON, C.G. & WHITE, D.E. (1987). A progress report on the treatment of 157 patients with advanced cancer using lymphokine-activated killer cells and interleukin-2 or high-dose interleukin-2 alone. *N. Engl. J. Med.*, **316**, 889–897.

ROSENBERG, S.A., LOTZE, M.T., YANG, J.C., AEBERSOLD, P.M., LINEHAN, W.M., SEIPP, C.A., WHITE, D.E. (1989*a*). Experience with the use of high dose interleukin-2 in the treatment of 652 cancer patients. *Ann. Surg.*, **210**, 474–485.

ROSENBERG, S.A., LOTZE, M.T., YANG, J.C., LINEHAN, W.M., SEIPP, C., CALABRO, S., KARP, S.E., SHERRY, R.M., STEINBERG, S. & WHITE, D.E. (1989*b*). Combination therapy with interleukin-2 and alpha-interferon for the treatment of patients with advanced cancer. *J. Clin. Oncol.*, **7**, 1863–1874.

SIEGEL, J.P. & PURI, R.K. (1991). Interleukin-2 toxicity. *J. Clin. Oncol.*, **9**, 694–704.

SLEIJFER, D., JANSSEN, R., BUTER, J., DE VRIES, E.G.E., WILLEMSE, P.H.B. & MULDER, N.H. (1992). Phase II of subcutaneous interleukin 2 in unselected patients with advanced renal cell carcinoma on an outpatient basis. *J. Clin. Oncol.*, **10**, 1119–1123.

STEIN, R.C., MALKOVSKA, V., MORGAN, S., GALAZKA, A., ANISZEWSKI, C., ROY, S.E., SHEARER, R.J., MARSDEN, R.A., BEVAN, D., GORDON-SMITH, E.C. & COOMBES, R.C. (1991). The clinical effects of prolonged treatment of patients with advanced cancer with low-dose subcutaneous interleukin 2. *Br. J. Cancer*, **63**, 275–278.

VON DER MAASE, H., GEERTSEN, P., THATCHER, N., JASMIN, C., MERCATELLO, A., FOSSA, S.D., SYMANN, M., STOTER, G., NAGEL, G., ISRAEL, L., OSKAM, R., PALMER, P. & FRANKS, C.R. (1991). Recombinant interleukin-2 in metastatic renal cell carcinoma. A European multicentre phase II study. *Eur. J. Cancer. Clin. Oncol.*, **27**, 1583–1589.

WEST, W.H., TAUER, K.W., YANNELLI, J.R., MARSHALL, G.D., ORR, D.W., THURMAN, G.B. & OLDHAM, R.K. (1987). Constant-infusion recombinant interleukin-2 in adoptive immunotherapy of advanced cancer. *N. Engl. J. Med.*, **316**, 898–905.

WHITEHEAD, R.P., WARD, D., HEMINGWAY, L., HEMSTREET III, G.P., BRADLEY, E. & KONRAD, M. (1990). Subcutaneous recombinant interleukin 2 in a dose escalating regimen in patients with metastatic renal cell adenocarcinoma. *Cancer Res.*, **50**, 6708–6715.