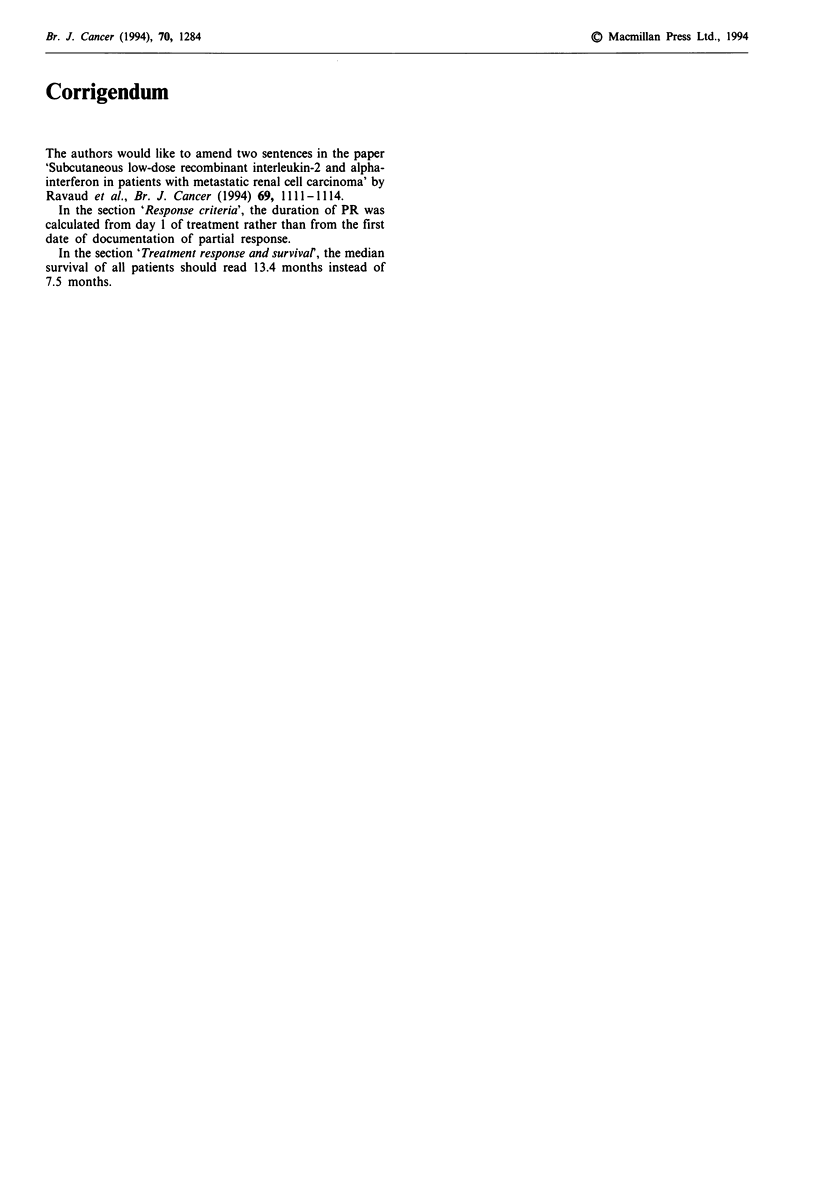# Corrigendum

**Published:** 1994-12

**Authors:** 


*Br. J. Cancer* (1994), **70**, 1284 © Macmillan Press Ltd., 1994

### Corrigendum

The authors would like to amend two sentences in the paper 'Subcutaneous low-dose recombinant interleukin-2 and alpha-interferon in patients with metastatic renal cell carcinoma' by Ravaud *et al*., *Br. J. Cancer* (1994) **69**, 1111–1114.

In the section '*Response criteria*', the duration of PR was calculated from day 1 of treatment rather than from the first date of documentation of partial response.

In the section '*Treatment response and survival*', the median survival of all patients should read 13.4 months instead of 7.5 months.